# Fluorescent labelling of the actin cytoskeleton in plants using a cameloid antibody

**DOI:** 10.1186/1746-4811-10-12

**Published:** 2014-05-19

**Authors:** Alessandra Rocchetti, Chris Hawes, Verena Kriechbaumer

**Affiliations:** 1 Biological and Medical Sciences, Oxford Brookes University, Oxford OX3 0BP, UK

**Keywords:** Actin, Nanobody, Chromobody, Golgi body, Actin dynamics

## Abstract

**Background:**

Certain members of the *Camelidae* family produce a special type of antibody with only one heavy chain. The antigen binding domains are the smallest functional fragments of these heavy-chain only antibodies and as a consequence have been termed nanobodies. Discovery of these nanobodies has allowed the development of a number of therapeutic proteins and tools.

In this study a class of nanobodies fused to fluorescent proteins (chromobodies), and therefore allowing antigen-binding and visualisation by fluorescence, have been used. Such chromobodies can be expressed in living cells and used as genetically encoded immunocytochemical markers.

**Results:**

Here a modified version of the commercially available Actin-Chromobody® as a novel tool for visualising actin dynamics in tobacco leaf cells was tested. The actin-chromobody binds to actin in a specific manner. Treatment with latrunculin B, a drug which disrupts the actin cytoskeleton through inhibition of polymerisation results in loss of fluorescence after less than 30 min but this can be rapidly restored by washing out latrunculin B and thereby allowing the actin filaments to repolymerise.

To test the effect of the actin-chromobody on actin dynamics and compare it to one of the conventional labelling probes, Lifeact, the effect of both probes on Golgi movement was studied as the motility of Golgi bodies is largely dependent on the actin cytoskeleton. With the actin-chromobody expressed in cells, Golgi body movement was slowed down but the manner of movement rather than speed was affected less than with Lifeact.

**Conclusions:**

The actin-chromobody technique presented in this study provides a novel option for *in vivo* labelling of the actin cytoskeleton in comparison to conventionally used probes that are based on actin binding proteins.

The actin-chromobody is particularly beneficial to study actin dynamics in plant cells as it does label actin without impairing dynamic movement and polymerisation of the actin filaments.

## Background

### Expression and applications of antibody constructs

In 1989, a novel type of antibody was identified first in the sera of dromedaries and later on in various members of the *Camelidae* family [1]. These antibodies differ from the typical antibody composition of two heavy and two light chains in that they are composed of just one heavy chain. Camelids produce both conventional and heavy-chain only antibodies (HcAbs) in ratios differing by species; 45% of llama serum antibodies are HcAbs and 75% in camels [1]. Isolation of the antigen binding domain (V_H_H, variable heavy chain of a heavy-chain antibody), the smallest functional fragment of these heavy-chain only antibodies, called nanobodies, lead to the development of various therapeutic proteins and tools.

Antibodies have the potential to bind to and therefore detect any molecule and cell structure making them a powerful research tool. Nanobodies only have a molecular mass of around 13 kDa and a size of 2 nm × 4 nm [2,3]. This small size offers several advantages over conventional antibodies or even antibody fragments such as monovalent antibody fragments (Fab) and single-chain variable fragments (scFv). For instance, for expression studies, only one protein domain has to be cloned and expressed. Nanobodies also show high stability and solubility even at high temperatures and under denaturing conditions [4,5]. Due to their stable and soluble nature, plus small size, high levels of expression are possible in heterologous systems in a reproducible manner and such features also allow for fusions to fluorescent proteins or protein tags [6]. Specific nanobodies can be screened for in a phage display system [7]. Nanobodies have been shown to be produced and functional in cellular compartments and environments that do not allow formation of disulphide bonds and are therefore functional in living cells [8]. In contrast to the flat or concave antigen binding site of conventional antibodies nanobodies display a convex conformation [9,3], allowing binding into otherwise inaccessible clefts and pockets which has proven a useful tool for inhibiting specific molecules such as lysozyme enzymes [9]. Furthermore, nanobodies still show binding affinities, like scFvs, in the nanomolar or even picomolar range [5].

Nanobodies have been used and tested in various applications. For instance they are considered for inhibitory therapeutic applications against viruses such as Influenza A, Respiratory Syncytial virus and Rabies virus [10] or even HIV-1 [11,12] to name a few [reviewed in [13].

A growing tool for manipulating animal and plant systems is the use of antibodies not only for inhibiting but altering the function of molecules. Nanobodies are the system of choice for such due to their ability to function intracellularly. In potatoes it was shown that they can target to the correct organelle and inhibit the function of the potato starch branching enzyme A more efficiently than an antisense construct [14]. A recent application of nanobodies has been the detection of the castor bean plant toxin ricin, a notorious bioterrorism agent. The nanobodies not only show high sensitivity towards ricin but also high specificity in distinguishing ricin from the non-toxic castor bean protein RCA120 [15].

The class of biomarkers used in this study have been termed “chromobodies” as they consist of nanobodies fused to fluorescent proteins generating fluorescent antigen-binding nanobodies that can be expressed in living cells [16]. Chromobodies have been shown to be useful tools in the real-time detection of dynamic changes in chromatin, nuclear lamina and the cytoskeleton in animal cells [16]. Such fusions have been shown to label and visualise endogenous cellular structures without disturbing cellular functions allowing real time studies of live cells processes [16].

### Actin cytoskeleton

The actin-cytoskeleton in animal cells is central to cell shaping, polarity and motility [17]. Most, but not all, plant cells contain a vacuole occupying up to 90% of the intercellular volume and are caged into a rigid cell wall limiting the cell expansion [18]. The cytoplasm is therefore constrained to a thin layer at the cell cortex and the actin-cytoskeleton sustains both the organisation of the cortical endomembrane system and cytoplasmic streaming [19,20].

The actin cytoskeleton is a network composed of fine 7 nm diameter filaments that can form bundles. It is continuously rearranging and actin dynamics have been described according to a stochastic model: filaments rapidly elongate at the barbed end, change shape, slide one along the other to bundle and finally break down [21]. Actin bundles and fine filaments have different fluorescence intensity when labelled as well as differences in resistance to depolymerising agents and dynamics. Bundles are brighter, more stationary over time and depolymerise more slowly; the latter have faint fluorescence, are more dynamic and can depolymerise rapidly [22].

Different labelling strategies have been developed to study the organisation and dynamics of actin filaments in plants. The expression of fluorescent actin has not proved useful in plants because most of it stays in monomeric form and diffuse in the cytoplasm resulting in a strong fluorescent background [23]. Phalloidin, a toxin extracted from death cup *Amanita phalloides*, binds and stabilizes F-actin and when conjugated to the fluorescent dye rhodamine selectively stains actin filaments in permeabilised and fixed plant cells. Rhodamine-phalloidin staining is also effective in unfixed cells but favours the formation of bundles [24]. As such it is not useful for any study of actin dynamics.

Actin binding proteins (ABPs) are involved in regulating the assembly of actin filaments and therefore are good marker candidates [25]. The actin binding domain of different ABPs have been fused to fluorescent proteins and expressed in plants. Lifeact, the most recently developed probe, is a 17 amino acid peptide from the yeast protein Abp140 that decorates F-actin [26]. In *Arabidopsis thaliana* Lifeact fused to the fluorescent protein Venus affects the reorganisation rate of bundles and cytoplasmic strands of the actin cytoskeleton at higher expression levels but has proven to be most valuable at optimised lower expression levels as it is currently the best probe to labels dynamic populations of actin filaments [27]. The actin binding domain of mouse talin fused to fluorescent proteins has been used to label plant actin filaments but has severe effects on the actin cytoskeleton and its depolymerisation [28]. One of the two actin-binding domains of the *A. thaliana* fimbrin1 protein (AtFIM1) fused to GFP (GFP-fABD2) labels the fine actin dynamic scaffold in different species and cell types. Stable expression in *A. thaliana* did not show adverse effects on general morphology or development [29].

All of the fluorescent reporters available so far depict varying organisations of the actin network. This may be due to a preferential binding to fine actin filaments rather than bundles or because the marker is derived from an actin-bundling protein therefore causing the aggregation of actin filaments. Considering that the actin cytoskeleton is a continuously re-arranging scaffold that provides tracks for movement and positioning of organelles such as Golgi bodies [30], a more reliable and less interfering fluorescent marker is needed for *in vivo* imaging.

In this study we used a modified version of the commercially available Actin-Chromobody® (ChromoTek, Martinsried, Germany) as a novel tool for visualising actin dynamics in tobacco leaf cells. The originally supplied plasmid contains the 13 kDa actin-binding alpaca V_H_H fused to a C-terminal GFP protein. This chromobody was previously used to transfect HeLa cells to show the recovery of the actin filaments after Cytochalasin D treatment (ChromoTek homepage) where it was shown that the transient binding does not influence cell viability or motility.

## Results and discussion

### In planta expression of the actin-chromobody

**Figure 1:**
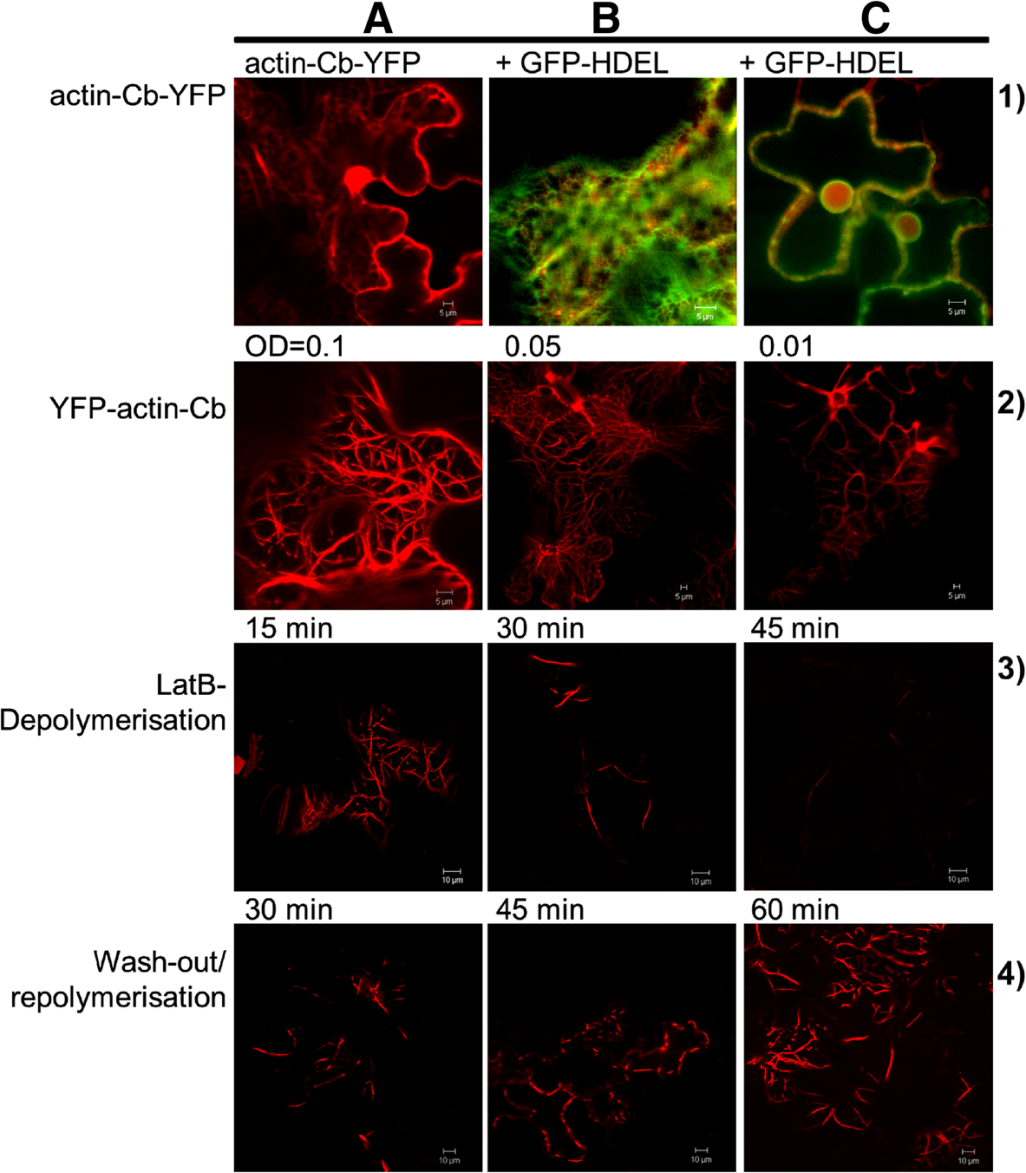
**Transient expression of the actin-chromobody.** Lane 1) Transient expression of the actin-chromobody (Actin-Cb) construct with a C-terminal YFP-fusion in *Nicotiana tabacum* leaves **(A)**; co-expression with the endoplasmic reticulum marker HDEL fused to GFP is shown **(B, C)**. Lane 2) Transient expression of the actin-chromobody (actin-Cb) construct with an N-terminal YFP-fusion in *Nicotiana tabacum* leaves at different Agrobacterium concentrations: OD_600_ = 0.1 **(A)**, OD_600_ = 0.0.5 **(B)**, OD_600_ = 0.01 **(C)**. Lane 3) Depolymerisation of actin cytoskeleton (labelled by the actin-chromobody) after 15 in **(A)**, 30 min **(B)** and (45 min **(C)** treatment with 25 μM latrunculin B (LatB). Lane 4) Repolymerisation of the actin cytoskeleton by washing out the LatB after 30 min **(A)**, 45 min **(B)** and 60 min **(C)**, respectively.

Constructs fusing the antibody sequence with both N- and C-terminal fluorescent protein tags, respectively, were prepared. *Agrobacterium tumefaciens* was transformed with these constructs and *Nicotiana tabacum* leaves were infiltrated with the transformed agrobacteria, either singly or with the Golgi marker consisting of the signal anchor sequence of a rat sialyl transferase fused to GFP [ST-GFP, [31] as described in [32]. In mammalian cells the C-terminal fusion expressed and actin targeting of the chromobody was reported (http://www.chromotek.com/products/chromobodies/actin-chromobody), whereas in plant cells the antibody C-terminal fusion remained cytosolic (Figure 1, lane 1A) with no fluorescence in other organelles such as the endoplasmic reticulum (Figure 1, lane 1B) but was found in the nucleoplasm (Figure 1, lane 1C), which is common for cytosolic proteins [33]. The N-terminal YFP-fusion, however, clearly labelled actin filaments in a specific manner (Figure 1, lane 2A-C).

**Figure 2:**
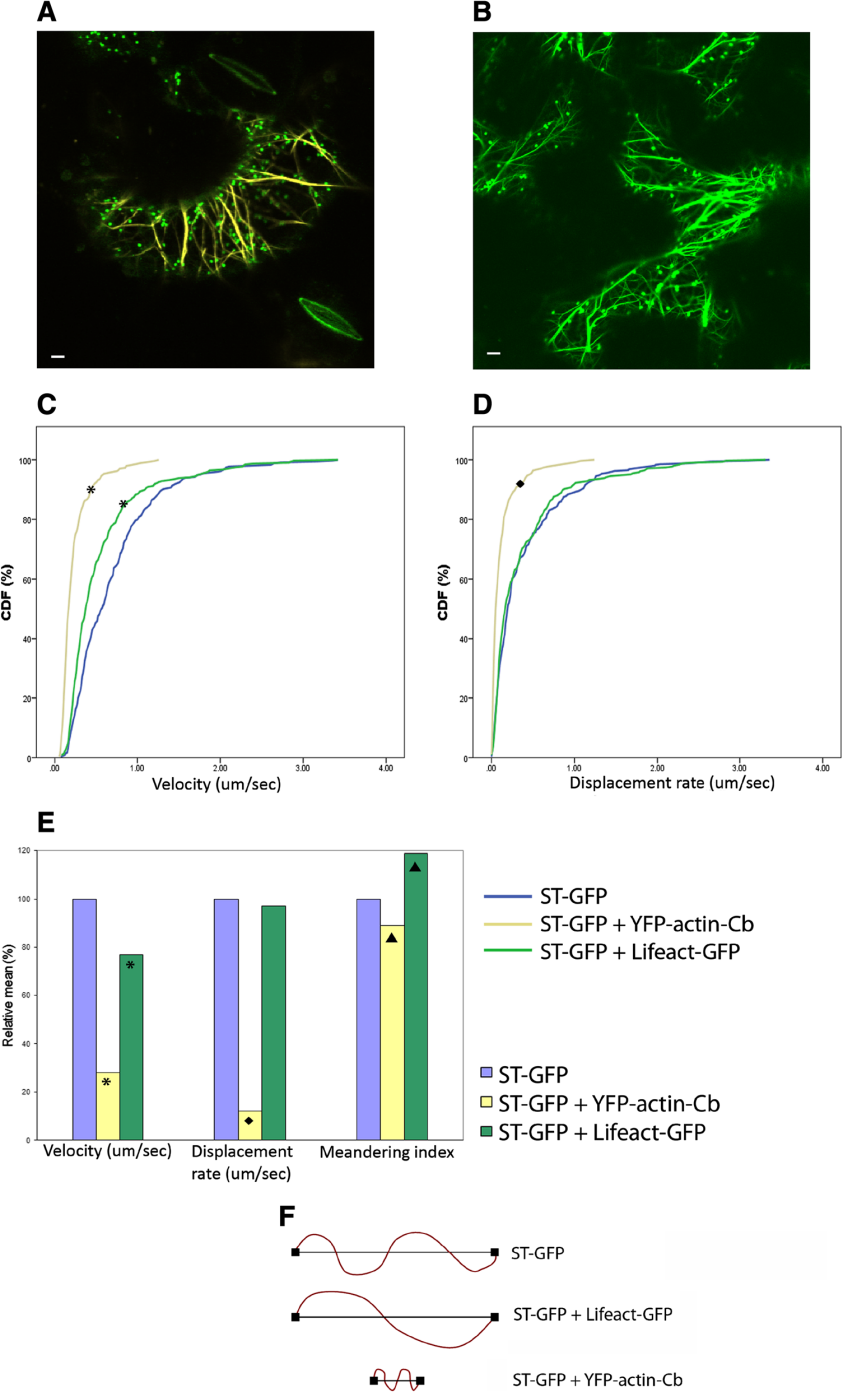
**Golgi movement and actin cytoskeleton dynamics. A)** Transient co-expression of the actin-chromobody (YFP-actin-Cb in yellow) construct with the Golgi marker ST-GFP (green dots); **B)** co-expression of Lifeact GFP (green lines) with ST-GFP (green dots). Scale bars = 5 μm. Cumulative Distribution Frequency (CDF) plots of velocity **(C)** and displacement rate **(D)** of *N. tabacum* transiently expressing only ST-GFP (blue line), both ST-GFP and YFP-actin-Cb (yellow line) or ST-GFP and Lifeact-GFP (green line), respectively. Curves marked with shapes (*, ♦) indicate a statistically significant difference from the control ST-GFP of p < 0.05. **E)** Velocity, displacement rate and meandering index values calculated with Volocity software for *N. tabacum* transiently expressing ST-GFP (blue line), both ST-GFP and YFP-actin-Cb (yellow line) or ST-GFP and Lifeact-GFP (green line), respectively. Mean values are expressed as a percentage of the control (ST-GFP). Symbols (*, ♦, ▲) indicate that the means are significantly different from the control at p < 0.05. **F)** Schematic representation of the path and movement pattern of Golgi bodies. When ST-GFP is coexpressed with Lifeact-GFP, Golgi bodies move same linear distance as the control but have a less salutatory path. The coexpression of the antibody determines Golgi bodies moving shorter linear distance and slightly more salutatory.

To determine optimal expression conditions that would allow investigation of actin dynamics as well as provide sufficient expression levels for visualisation, tobacco leaves were infiltrated with three different concentrations of *Agrobacterium tumefaciens*: OD_600_ of 0.1, 0.05 and 0.01 with 0.1 being the conventional infiltration OD. The highest OD of 0.1 resulted in major bundling of actin filaments (Figure 1, lane 2A) and at the lowest OD of 0.01 the construct mainly bound to thicker actin bundles (Figure 1, lane 2C). The OD of 0.05 labelled both thicker filaments as well as finer ones (Figure 1, lane 2B) and was therefore chosen for follow-up experimentation. In general at OD 0.05 what appeared to be a more complete overview of the actin cytoskeleton with thick bundles and thinner filaments was obtained compared to that with Lifeact expression (Figure 2B). On coexpression with the ST-GFP Golgi marker, Golgi bodies could clearly be seen to be associated with the actin filament bundles as previously reported [31].

To investigate if actin dynamics was impaired by the chromobody binding leaf segments were treated with 25 μM latrunculin B (LatB), an agent isolated from a Red Sea sponge known to disrupt the actin cytoskeleton of cells. LatB binds monomeric actin with 1:1 stoichiometry and thereby blocks F-actin polymerisation without affecting cell viability [34]. After 15 min of LatB treatment the thinner strands were completely absent (Figure 1 lane 3A) and after 30 min and 45 min only the bundled actin strands were visible (Figure 1 lane 3B, C); these bundles remain even with overnight treatment in the drug (data not shown).

With LatB being a relatively small molecule of less than 0.4 kDa it is possible to reverse its effects by immersing the leaf cuttings in water and thereby washing out the drug and allowing the actin filaments to repolymerise. A rapid recovery of filamentous actin within less than one hour of washing was observed (Figure 1, lane 4B) with a visible increase in strands in 30 min (Figure 1, lane 4A).

### Impact of actin-chromobody labelling on actin function

The motility of Golgi bodies is mainly dependent on the actin cytoskeleton and treatment with the actin depolymerising agent cytochalasin D caused the organelles to stop moving [35]. However, *in vivo* labelling of the actin cytoskeleton might compromise the dynamics of the organelle changing the organisation of the actin network [27].

In order to compare the effect of Lifeact-GFP and the YFP-actin-Cb on the movement of Golgi bodies in *N. tabacum*, the cytoskeleton markers were transiently coexpressed with the Golgi marker ST-GFP (Figure 2A, B) and compared to the transient expression of ST-GFP only. For this, the infiltration OD and therefore the expression levels for Lifeact were chosen for optimal Golgi movement and as little bundling as possible. Movies were collected for each combination and analysed with Volocity software to track Golgi bodies and calculate parameters such as velocity, displacement rate and meandering index. The values are represented as Cumulative Distribution Frequency (CDF) and means are normalised against data from ST-GFP expression alone.

The expression of either the cytoskeleton markers significantly slowed the motility of Golgi bodies as described by their velocity which is the length of the track over time (Figure 2C). The displacement rate, which is the linear distance between the initial and final position of the organelle, was not significantly affected by the expression of Lifeact-GFP but was significantly reduced in the presence of YFP-actin-Cb (Figure 2D). Both velocity and displacement rate in the combinations of ST-GFP and YFP-actin-Cb or Lifeact-GFP, respectively, were normalised to the datasets for expression of ST-GFP alone and presented as a percentage of the latter (Figure 2E). The final parameter assessed was the Meandering Index which is the ratio between the displacement rate and velocity (the ratio between the linear distance and the overall path of the Golgi body), describing the type of Golgi movement. The closer the meandering index is to a value of 1, the more directional and linear is the movement. The smaller the meandering index, the more saltatory is the organelle movement. The meandering index therefore gives an indication of the complexity of the dynamics. Upon the expression of Lifeact-GFP, the relative mean of the meandering index was significantly increased by 19% compared to ST-GFP alone indicating that the organelles moved more uni-directionally (Figure 2E). YFP-actin-Cb significantly reduced the meandering index by 11% with respect to ST-GFP (Figure 2E) indicating that the saltatory movement was slightly favoured. The emerging model (Figure 2F) is that given a fixed time span of 1 second Golgi bodies move more slowly, with a shorter linear distance but conserve the complexity of the movement pattern in the presence of the antibody. Coexpression with Lifeact-GFP results in a reduction of the velocity, with the same linear distance but a much less saltatory movement.

These effects of the markers on actin dynamics might be explained by the different effects the two markers have on cytoskeleton rearrangement and thus its dynamic. Lifeact derives from a cross bundling factor and favours the formation of actin cables that might lead Golgi bodies along more directional pathways whilst binding of the actin-Cb might interfere less with the filament organisation therefore having a less of an effect on the movement type.

## Conclusion

Mainly due to their small size and stability in combination with production advantages, nanobodies have been shown to be valuable tools for inhibiting or manipulating cell processes with a great potential for genetically encoded *in vivo* immunocytochemical labelling.

The actin-chromobody described here is especially useful for the study of actin dynamics in plant cells as it labels the actin, but does not overall impair the pattern of organelle movement, although it does slow organelle velocity. It is thus another option for *in vivo* labelling of actin compared with the commonly used fluorescent protein probes based around actin binding proteins or their active domains. It may be possible to exploit the reduction in organelle movement and by implication myosin activity, for the study of organelle dynamics and their relationship with the actin cytoskeleton.

## Methods

### Cloning of expression plasmids

The Actin-Chromobody® plasmid containing the alpaca actin-antibody gene was obtained from ChromoTek (Martinsried, Germany). Primers were ordered from Eurofins MWG Operon (Ebersberg, Germany). Q5 high-fidelity DNA polymerase (New England Biolabs, Herts, UK) was used for all polymerase chain reaction (PCR) reactions. The actin-Ab-PCR product was cloned into the binary vectors PB7WGY2 and PB7YWG2 providing an N- or C-terminal YFP-tag, respectively, using Gateway® technology (Invitrogen life sciences).

### Plant material and transient expression system

For Agrobacterium-mediated transient expression, 4-week-old tobacco (*Nicotiana tabacum* SR1 cv Petit Havana) plants grown in the greenhouse were used. Briefly, each expression vector was introduced into Agrobacterium strain GV3101 (pMP90) by heat shock. A single colony from the transformants was inoculated into 5 ml of YEB medium (per litre: 5 g of beef extract, 1 g of yeast extract, 5 g of sucrose and 0.5 g of MgSO_4_·7H_2_O) supplemented with 50 μg/ml spectinomycin and rifampicin. After overnight shaking at 25°C, 1 ml of the bacterial culture was pelleted in a 1.5-ml tube by centrifugation at 2200 × g for 5 min at room temperature. The pellet was washed twice with 1 ml of infiltration medium (50 mM MES, 2 mM Na_3_PO4·12H_2_O, 0.1 mM acetosyringone and 5 mg/ml glucose) and then resuspended in 1 ml of infiltration buffer. The bacterial suspension was diluted with the same buffer to adjust the inoculum concentration to the desired final OD_600_ (0.1, 0.05 or 0.01) for YFP-actin-Cb and OD_600_ = 0.01 for Lifeact-GFP and inoculated using a 1 ml syringe without a needle by gentle pressure through the stomata on the lower epidermal surface. For experiments requiring co-infection of more than one construct, bacterial strains containing the constructs were mixed prior to the leaf infiltration, with the inoculum of each mixed construct adjusted to the required final OD_600_. Transformed plants then were incubated under normal growth conditions for 48 h.

Images were taken using a Zeiss LSM510 Meta laser scanning confocal microscope (http://www.zeiss.com/) with 40× and 63× oil immersion objectives. For imaging of GFP/YFP combinations, samples were excited using 458 and 514 nm laser lines in multi-track mode with line switching. Images were edited using the LSM510 image browser and Adobe Photoshop.

### Microscopy and movies

For dual imaging 488 nm excitation and 505-530 band pass filters were used for eGFP and for YFP an excitation of 514 nm and BP 470-500 was used; dual imaging of GFP and YFP was captured as described above.

Movies were acquired using 63X objective lens, zoomed to ×3.7 and a ROI of 244 × 244 pixels. Movies of 50 frames were acquired at scan time of 470 msec. Example movie files used for this analysis are shown in Additional file 1.

### Organelle tracking and statistical analysis

Organelle tracking was done using the Volocity 6.3 (Improvision - PerkinElmer). Intensity and size parameters were set and the software identified and tracked Golgi bodies according to shortest path model. The velocity, displacement rate and meandering index of 100-344 Golgi bodies per condition were calculated by the software. Statistical analysis and graphs were done with SPSS 21.0 and the Kolmogorov-Smirnov test was used to assess the statistical difference in the distribution of velocity, displacement rate and meandering index values for p < 0.05.

## Abbreviations

HcAbs: Heavy-chain only antibodies; VHH: Variable heavy chain of a heavy-chain antibody; scFv: Single-chain variable fragments; ABP: Actin binding protein; GFP: Green fluorescent protein; YFP: Yellow fluorescent protein; ST: Rat sialyl transferase; LatB: Latrunculin B; actin-Cb: Actin chromobody; CDF: Cumulative Distribution Frequency.

## Competing interests

The authors declare that they have no competing interests.

## Authors’ contributions

AR participated in the study design, carried out expression studies, analysed Golgi dynamics and helped draft the manuscript. CH conceived of the study, and participated in its design and coordination and helped to draft the manuscript. VK participated in the study design, made the constructs for expression, carried out expression studies and helped draft the manuscript. All authors read and approved the final manuscript.

## Supplementary Material

Additional file 1 **Example movies of Golgi body movement.** Movement of Golgi bodies (ST-GFP labelled, green dots) in co-expression with YFP-actin-Cb (A) or Lifeact-GFP (B), respectively. Green fluorescent (Golgi) dots in movies were analysed with the Volocity software to calculate velocity, displacement rate and meandering index of Golgi body movement. Click here for file
